# Efficient CRISPR Mutagenesis in Sturgeon Demonstrates Its Utility in Large, Slow-Maturing Vertebrates

**DOI:** 10.3389/fcell.2022.750833

**Published:** 2022-02-10

**Authors:** Jan Stundl, Vladimír Soukup, Roman Franěk, Anna Pospisilova, Viktorie Psutkova, Martin Pšenička, Robert Cerny, Marianne E. Bronner, Daniel Meulemans Medeiros, David Jandzik

**Affiliations:** ^1^ Department of Zoology, Faculty of Science, Charles University, Prague, Czechia; ^2^ Division of Biology and Biological Engineering, California Institute of Technology, Pasadena, CA, United States; ^3^ South Bohemian Research Center of Aquaculture and Biodiversity of Hydrocenoses, Faculty of Fisheries and Protection of Waters, University of South Bohemia in České Budějovice, Vodňany, Czechia; ^4^ Department of Ecology and Evolutionary Biology, University of Colorado in Boulder, Boulder, CO, United States; ^5^ Department of Zoology, Faculty of Natural Sciences, Comenius University in Bratislava, Bratislava, Slovakia

**Keywords:** CRISPR/Cas9, targeted mutagenesis, non-teleost fish, sturgeon, vertebrates, development, evolution, evo-devo

## Abstract

In the last decade, the CRISPR/Cas9 bacterial virus defense system has been adapted as a user-friendly, efficient, and precise method for targeted mutagenesis in eukaryotes. Though CRISPR/Cas9 has proven effective in a diverse range of organisms, it is still most often used to create mutant lines in lab-reared genetic model systems. However, one major advantage of CRISPR/Cas9 mutagenesis over previous gene targeting approaches is that its high efficiency allows the immediate generation of near-null mosaic mutants. This feature could potentially allow genotype to be linked to phenotype in organisms with life histories that preclude the establishment of purebred genetic lines; a group that includes the vast majority of vertebrate species. Of particular interest to scholars of early vertebrate evolution are several long-lived and slow-maturing fishes that diverged from two dominant modern lineages, teleosts and tetrapods, in the Ordovician, or before. These early-diverging or “basal” vertebrates include the jawless cyclostomes, cartilaginous fishes, and various non-teleost ray-finned fishes. In addition to occupying critical phylogenetic positions, these groups possess combinations of derived and ancestral features not seen in conventional model vertebrates, and thus provide an opportunity for understanding the genetic bases of such traits. Here we report successful use of CRISPR/Cas9 mutagenesis in one such non-teleost fish, sterlet *Acipenser ruthenus*, a small species of sturgeon. We introduced mutations into the genes *Tyrosinase*, which is needed for melanin production, and *Sonic hedgehog*, a pleiotropic developmental regulator with diverse roles in early embryonic patterning and organogenesis. We observed disruption of both loci and the production of consistent phenotypes, including both near-null mutants’ various hypomorphs. Based on these results, and previous work in lamprey and amphibians, we discuss how CRISPR/Cas9 F0 mutagenesis may be successfully adapted to other long-lived, slow-maturing aquatic vertebrates and identify the ease of obtaining and injecting eggs and/or zygotes as the main challenges.

## Introduction

The central problem in modern biology is understanding how an organism’s one-dimensional genotype, i.e., its linear sequence of nucleotides, gives rise to its four-dimensional phenotype, i.e., its form and function through space and time. For decades, genotype was linked to phenotype by mapping genetic lesions in purebred lines of genetic model organisms. These organisms were carefully selected based on their rapid life cycles and ability to be easily maintained in the laboratory (Hedges, 2002). Mutations occurred either naturally, or were introduced randomly in the genome using radiation, chemicals, or transposable elements. In the 1980s and 1990s, various methods were developed that allowed targeted mutagenesis in select model organisms including *Drosophila, Caenorhabditis elegans*, and mouse (Robertson et al., 1988; Kaiser and Goodwin, 1990; Jansen et al., 1997; Faruqi et al., 1998). In the 2000s new “one-size-fits-all” gene targeting technologies, including TALENs and Zinc finger nucleases, allowed targeted mutagenesis in a greater variety of genetic models, such as zebrafish (Doyon et al., 2008; Meng et al., 2008; Huang et al., 2011; Wood et al., 2011). While effective, these methods still required the generation of purebred lines to determine the complete phenotype caused by the mutation. Thus, while substantially faster and more refined than previous methods, gene targeting was still largely limited to a handful of conventional genetic model systems. Furthermore, because these organisms were chosen specifically for their atypical life histories, our understanding of gene function was still largely limited to a few isolated twigs on the vast tree of life.

In the 2010s, these gene targeting strategies became largely supplanted by CRISPR/Cas9-mediated mutagenesis (Bassett et al., 2013; Chen et al., 2013; Fu et al., 2013; Hwang et al., 2013; Li-En et al., 2013; Nakayama et al., 2013; Waaijers et al., 2013; Port et al., 2014; Square et al., 2015; Véron et al., 2015; Martin et al., 2016; Suzuki et al., 2018; Rasys et al., 2019). The CRISPR/Cas9 method results in small, targeted lesions when the cell’s DNA repair mechanisms respond to double-stranded DNA breaks created by the Cas9 endonuclease (Barrangou et al., 2007; Jinek et al., 2012; Mali et al., 2013). Practically, CRISPR/Cas9 mutagenesis has several distinct advantages over TALENs and Zinc finger nucleases. Most significantly is its ease of use (Ran et al., 2013). Cas9 endonucleases are commercially available, and quickly and affordably programmed to cleave specific target sequences by binding short guide RNAs (sgRNAs) (Cong et al., 2013; Ran et al., 2013; Hsu et al., 2014). Another major advantage of CRISPR/Cas9 mutagenesis over previous gene targeting methods is its speed and efficiency (Mali et al., 2013; Ran et al., 2013; Fei et al., 2014). Target site cutting occurs minutes or hours after the Cas9-sgRNA complex enters the cytoplasm. In the case of zygotes, this means that the Cas9-sgRNA complex acts before and during early cell cleavage stages, creating mosaic mutant individuals (Mizuno et al., 2014; Yen et al., 2014). With highly efficient sgRNAs, most of the cells in these “F0” mosaic mutant individuals (sometimes called “crispants” (Burger et al., 2016)) will possess biallelic deletions in the targeted sequence (e.g. Blitz et al., 2013; Jao et al., 2013). CRISPR/Cas9 mutagenesis is also extremely versatile (Mali et al., 2013; Hsu et al., 2014). Despite evolving as a component of the prokaryotic adaptive immune system (Barrangou et al., 2007; Deveau et al., 2010; Garneau et al., 2010; Horvath and Barrangou, 2010; Makarova et al., 2011), Cas9 endonuclease appears to function efficiently and specifically in any cell type regardless of species. Thus, CRISPR/Cas9 allows the rapid creation of near-null mutants in virtually any organism whose eggs and/or zygotes are amenable to injection with proteins and RNA.

The efficiency and versatility of CRISPR/Cas9 means that genotype and phenotype can now be linked in organisms not suitable for the establishment of purebred lines. Thus, gene function can now be studied in organisms chosen for features aside from their ability to be lab-reared, including phylogenetic position, possession of derived phenotypes, or similarity to ancestral forms (Stolfi et al., 2014; Square et al., 2015; Trible et al., 2017; Rasys et al., 2019; Crawford et al., 2020; Kiyonari et al., 2021; Mori and Nakamura, 2021). This has opened up the possibility of side-by-side comparisons of gene function across diverse taxa, allowing researchers to more easily deduce ancestral gene functions, and identify the genes underlying novel phenotypes. CRISPR/Cas9 mutagenesis has become a powerful tool for understanding the evolution of genes, genomes, and phenotypes across both large and small evolutionary timescales (Komor et al., 2017).

CRISPR/Cas9 mutagenesis is having a large impact on the study of vertebrate evolution (e.g. Barske et al., 2020; Square et al., 2020; Hawkins et al., 2021). This is because the majority of vertebrates are large, long-lived, slow-maturing organisms, and do not survive well in small laboratory enclosures. This is especially true for taxa that diverged from the two dominant modern lineages, teleosts and tetrapods, in the Ordovician, or before. These so-called “basal” fish include the living jawless fish, hagfish and lamprey, and non-teleost jawed fish such as sturgeon, paddlefish, gar, bichir and bowfin. Unlike zebrafish and mouse, these vertebrates are typically large, with long generation times and extended predatory adulthoods.

Extant sturgeons are the few remaining representatives of once-diverse radiation of non-teleost ray-finned fish, the order acipenseriforms, that diverged from the lineage leading to modern teleosts about 345 million years ago (Hughes et al., 2018; Du et al., 2020). Because of their phylogenetic position, comparisons between sturgeons and teleost models, like zebrafish and medaka, can provide insights into the biology of early ray-finned fish (e.g. Minarik et al., 2017; Stundl et al., 2020). In addition, Acipenseriforms possess a combination of ancestral and derived vertebrate traits not seen in teleosts, including an endoskeleton lacking proper bone, a body armor made of bony scutes, a heterocercal caudal fin, and a lack of teeth in adulthood (Bemis et al., 1997). Several species are also polyploid (Havelka et al., 2013; Rajkov et al., 2014; Symonová et al., 2017) and roe of some is considered a delicacy (Bemis et al., 1997). Together, these characters make sturgeons an object of study for scientists from diverse specializations, from genetics and genomics to developmental and evolutionary biology, to aquaculture and food production. Despite this broad interest, understanding the genetic bases of sturgeon traits is difficult because they are large, long-lived, slow-maturing, and poorly suited for the establishment of purebred lines.

**FIGURE 1:**
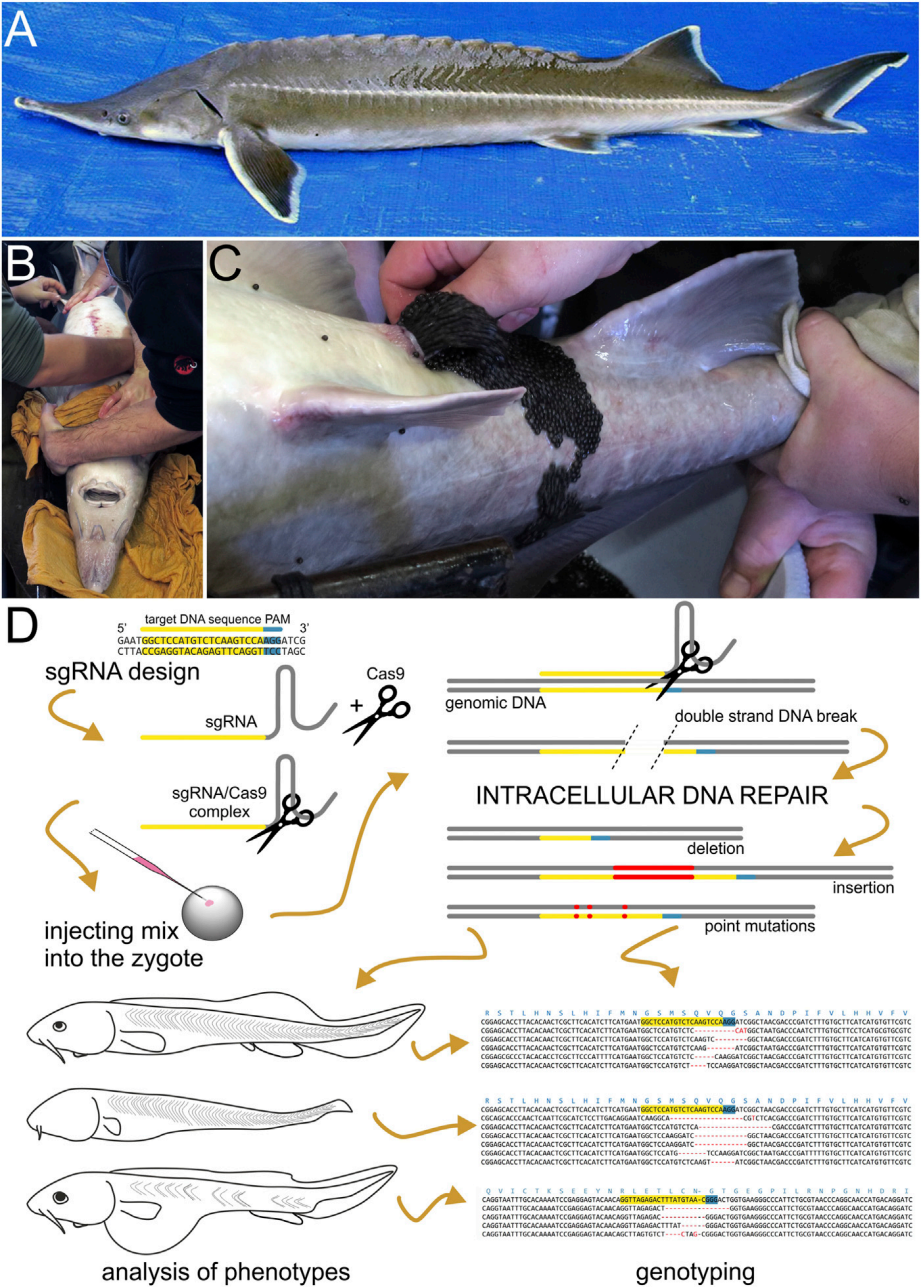
Sturgeon and CRISPR/Cas9 mutagenesis pipeline. **(A)** Dorsoleteral view of an adult individual of sterlet (*Acipenser ruthenus*). Abdomen of an adult anesthetized sturgeon female (pictured here is the Siberian sturgeon, *Acipenser baerii*) in dorsal recumbency **(B)** is manually massaged in anterior-to-posterior direction and ovulated eggs are collected into a bowl **(C)**. **(D)** A scheme illustrating the sterlet CRISPR/Cas9 mutagenesis pipeline. First, the single guide RNA (sgRNA) is designed and synthesized based on the selected target DNA sequences and next to Protospacer Adjacent Motif (PAM). PAM is required for proper function of Cas9 nuclease and is not part of sgRNA. Next, the sgRNA is mixed with Cas9 protein (illustrated here as scissors) to form a sgRNA/Cas9 complex. This mix is injected into sterlet zygotes. Cas9 nuclease navigated to the target by sgRNA breaks the double strand of DNA, which is subsequently repaired inside the cleaving cells and nuclei. (mainly by non-homologous end joining) The imperfect DNA repairs introduce mutations into newly synthesized molecules of DNA resulting into a mosaic of cells bearing DNA with indels and/or point mutations at the targeted site. Embryos and/or raised larvae are later inspected for phenotypic effects and selected candidates are genotyped.

We recently adapted the CRISPR/Cas9 method to the sea lamprey, *Petromyzon marinus*, and the African clawed frog, *Xenopus laevis* (Square et al., 2015; Square et al., 2020), to better understand the ancestral functions of vertebrate developmental regulatory genes. Here we report the successful application of the same strategy to the non-teleost jawed fish sterlet, *Acipenser ruthenus,* a small species of sturgeon (Figure 1A). We then discuss specific variables and general considerations for workers seeking to apply the CRISPR/Cas9 mutagenesis method to other non-teleost fish, or any aquatic vertebrate for which establishing purebred lines is difficult or impossible.

## Materials and Methods

### Animal Husbandry and *in vitro* Fertilization

We obtained the zygotes of sterlet (*Acipenser ruthenus* Linnaeus, 1758) from the adults kept and regularly bred at the Research Institute of Fish Culture and Hydrobiology, Faculty of Fisheries and Protection of Waters, University of South Bohemia in České Budějovice, Vodňany, Czech Republic (RIFCH). The husbandry, animal conditioning, gamete collection, and fertilization were described in detail by Chebanov and Galich (2011) and Saito et al. (2014). The adult breeding fish were handled under anesthesia in 0.05% tricaine. Briefly, both female and male adult sterlets, aged five to 9 years, were transferred from the outdoor ponds into 4,000 L indoor tanks with water temperature kept at constant 15°C. Spermiation in males was induced by intramuscular injection of carp pituitary extract at 4 mg/kg body weight in 0.9% NaCl solution. Sperm was collected 48 h later via a 0.6 mm catheter into cell culture flasks and kept on ice until *in vitro* fertilization. Female ovulation was induced in a similar way, but with two doses of the hormone instead of only one injected 12 h apart −0.5 and 4.5 mg/kg of body weight, respectively. Females ovulated 18–20 h after the hormone was administered. Ovulating females were placed in dorsal recumbency position and oviduct incision was performed using an eye microsurgery scalpel. This minimal invasive procedure allows accessing the ovulated eggs in sturgeon oviduct that is physiologically folded (see Chebanov and Galich, 2011). Then the female’s abdomen was massaged in anterior-to-posterior direction and eggs were collected into bowls (Figures 1B,C), sealed with aluminum foil and subsequently fertilized with sperm at 15°C in dechlorinated tap water. We only used sperm with the spermatozoa motility assessed at >80% (Chebanov and Galich, 2011). The zygotes were rinsed in 0.04% tannic acid to make them less adherent (Saito et al., 2014), and then kept in water at 15°C before they were used in experiments. After the injections, we transferred the embryos to 48-well plates, each in 1 ml volume of E2 zebrafish medium (Brand et al., 2002) containing antibiotics (120 ng/ml of penicillin and 200 ng/ml of streptomycin) kept at 15°C. The medium was changed daily or more often, if necessary. Embryos selected for raising to later developmental stages were transferred to well-oxygenated tanks with E2/antibiotics at 15–17°C shortly after they hatched, which typically happened 7 days post fertilization (st. 35). We staged the embryos and larvae using the staging system of Detlaff et al. (1993). When the embryos and larvae reached the desired developmental stage, they were anesthetized by tricaine (MS-222; Serva) and fixed in 4% PFA in PBS overnight at 4°C. After several washes in PBS, we gradually dehydrated the embryos and larvae through a series of PBS/methanol solutions and stored in 100% methanol at −20°C until further use.

Animal care and all experiments were approved by the Ministry of Agriculture of the Czech Republic (MSMT-12550/2016-3), followed the principles of the European Union Harmonized Animal Welfare Act of the Czech Republic, and Principles of Laboratory Animal Care and National Laws 246/1992 “Animal Welfare”, and were conducted in accordance with the Animal Research Committee of RIFCH. Authors of the study own the Certificate of professional competence for designing experiments and experimental projects under Section 15 d (3) of the Czech Republic Act no. 246/1992 Coll. on the Protection of Animals against Cruelty.

### Identification of Endogenous *Tyr* and *Shh* Loci in Sterlet Genome and Design of the sgRNAs

We used similar F0 mutagenesis strategy as was described for sea lamprey (*Petromyzon marinus*) and African clawed frog (*Xenopus laevis*) (Square et al., 2015; Square et al., 2020) (Figure 1D). Using the spotted gar (*Lepisosteus ocelatus*) and zebrafish (*Danio rerio*) tyrosinase and sonic hedgehog protein sequences as queries we searched sterlet *Tyr* and *Shh* homologs in our *de novo* assembled transcriptomes of sterlet pharyngulae (available online at https://www.researchgate.net/profile/David-Jandzik/projects). The identity of recovered sequences was checked with BLAST (Altschul et al., 1990). We targeted the protein-coding exons at loci showing high evolutionary conservation across vertebrate taxa. In both *tyr* and *shh* we identified four putative exons. We selected the best CRISPR/Cas9 target sites with sequence 5′-GG (18N) NGG-3′ and with no off-target matches to our transcriptomes. The individual sequences were visually checked and identified as off-targets if they showed more than 85% similarity to our candidate sequence including PAM by BLAST (0–3 mismatches) and the mismatches were close to PAM site (more than one mismatch closer to PAM than 10 bp). The sequences of target sites were as follows: *tyr* sgRNA 3: GGT​TAG​AGA​CTT​TAT​GTA​AC (GGG), *tyr* sgRNA 4: GGC​TCC​ATG​TCT​CAA​GTC​CA(AGG), *shh* gRNA1: (CCC)CAA​TGT​GGC​CGA​GAA​GAC​CC, *shh* gRNA2: GGG​CCA​GTG​GCA​GAT​ATG​AA(GGG) with PAM sites in parentheses and the PCR primers used to amplify the target sequences in Table 1. The amplified fragments were annealed and *in vitro* phosphorylated with T4 Polynuclease Kinase (NEB M0201S) at 37°C for 1h, and ligated into the DR274 plasmid (Hwang et al., 2013) pre-digested with BsaI (NEB R0535). Single guide RNAs were *in vitro* transcribed using T7 High Yield Kit (New England Biolabs) and purified by phenol-chloroform extraction followed by precipitation in 70% ethanol with 0.3M sodium acetate. The precipitate was resuspended in nuclease-free water.

**TABLE 1:** Primers used A) to amplify the DNA templates for sgRNAs synthesis, B) for genotyping the putative mutants, and C) to amplify the DNA template for *foxD3, ripply3*, and *twist1* RNA *in situ* hybridization probe.

Target	Forward primer	Reverse primer
A)		
*tyr* sgRNA 3	TAG​GTT​AGA​GAC​TTT​ATG​TAA​C	AAA​CGT​TAC​ATA​AAG​TCT​CTA​A
*tyr* sgRNA 4	TAG​GCT​CCA​TGT​CTC​AAG​TCC​A	AAA​CTG​GAC​TTG​AGA​CAT​GGA​G
*shh* sgRNA 1	TAG​GGT​CTT​CTC​GGC​CAC​ATT​G	AAA​CCA​ATG​TGG​CCG​AGA​AGA​C
*shh* sgRNA 2	TAG​GGC​CAG​TGG​CAG​ATA​TGA​A	AAA​CTT​CAT​ATC​TGC​CAC​TGG​C
B)		
*tyr* sgRNA 3 geno	GGA​GGA​AGC​AAA​CAA​CAT​AAG​CTA​CAG	CAC​GGA​TAT​GAC​TGG​AGG​TAA​CAG​TC
*tyr* sgRNA 4 geno	GCA​GTT​TAC​TTT​GCT​GCA​TGT​GTG	CCA​CGT​GGC​TGT​CTA​TCG​GTG
*shh* sgRNA 1and2 geno	CTT​TGG​TGT​CCT​CTG​GGC​TG	GAG​CCT​GTC​AGC​CCC​AGT​G
C)		
*foxD3* ISH probe	GAYGTGGAYATCGAYGTGGT	CTSARRAARCTVCCGTTGTC
*ripply3* ISH probe	AGA​TGC​AAT​CCA​CGG​GCT​AC	GTG​GAT​TGT​CGC​TTG​CAC​AG
*twist1* ISH probe	GAAAWGWTGCARGANGAATC	TGVGATGYRGACATGGACCA

### Microinjection

We prepared a fresh injection mix on ice shortly before each injection session. The commercially produced recombinant Cas9 protein (PNA Bio Inc.) was resuspended per manufacturer’s instructions to the stock concentration of 1 mg/ml, aliquoted, and stored at −80°C. For a 6 μl injection mix we first incubated 1.6 μg of diluted Cas9 with 800 ng of total sgRNA for 10 min on ice, then brought the total volume up to 5.5 μl with nuclease-free water. Approximately 0.6 μl of 50 μg/μl lysinated Rhodamine-dextran (LRD; Invitrogen) in nuclease-free water was then added, resulting in a final LRD concentration of 5 μg/μl. One-cell-stage embryos were manually dechorionated using Dumont forceps and positioned in shallow holes in modeling clay in a Petri dish to facilitate their proper orientation and stability. The microinjection was performed either with mouth or manual injector (set to 100 hPa for 1 s) using microcapillary needles (Drummond Microcaps) pulled in a Narishige pc-10 puller (58°C with two weight elements; diameter 1.02 mm). The needle tip diameter was adjusted to allow to produce a ∼20 nl drop (approximately 1/7 of the sterlet zygote in diameter) containing around 2.67 ng of sgRNA and 5.3 ng Cas9 protein (in 1:2 wight ratio) in total. While injecting, we targeted the animal pole of the embryo at a ∼45° angle (Figure 1D). After injection the embryos were transferred to 48-well plates with E2/antibiotics at 15°C. We screened the embryos for LRD at the neurula stage (ca. st. 21). To control for mortality rates, we injected in parallel a few batches of embryos with a non-functional sgRNA at the same mix composition and concentrations as the experimental mixes. Each sgRNA was injected multiple times, in eggs from multiple clutches and by several authors of this study.

### Analysis of Phenotypes

We assessed the efficiency of *tyr* mutagenesis in injected larvae raised approximately to 16 mm of total length, when pigmentation is visibly present and conspicuous on the head and body of the wild-type individuals. Based on the severity of pigmentation reduction we scored the observed phenotypes in four categories as 0–25% reduction, 25–50% reduction, 50–75% reduction, and 75–100% reduction. We also checked the larvae for any non-specific malformations and deformities. The embryos injected with *shh* sgRNAs with Cas9 protein were fixed at the pharyngula stage (st. 28), when several organs regulated by hedgehog signaling have formed. These include heart, pre-oral gut, olfactory and optic placodes, first and second pharyngeal pouches, fore-, mid-, and hindbrain, somites and pronephros. Rather than scoring the global phenotypes of the embryos and comparing the severity of the phenotypes among different characters, we scored each structure separately, recording whether it was present or reduced/deformed. We also used *in situ* hybridization to visualize changes in expression patterns of neural crest markers *foxd3* and *twist1* and pharyngeal pouch formation marker *ripply3* in mutant embryos.

### Genotyping

To confirm mutations of targeted loci, we genotyped selected sterlet embryos and larvae showing variable phenotypes; at least two individuals per sgRNA. Due to higher percentage of individuals showing no obvious reduction in pigmentation, i.e. wild-type-looking phenotype in *tyr* sgRNA injected larvae, we also genotyped a few of those to obtain a better picture of *tyr* sgRNA efficiency. In *tyr* sgRNA injected larvae we used tail clips obtained from freshly euthanized larvae, while in *shh* mutants we used the whole embryos after we photographed and analyzed their phenotypes or gene expression patterns. We digested the tissue with Proteinase K (80 IU/ml; Sigma-Aldrich) in 1X PCR buffer at 55°C for 12 h. The obtained genomic DNA extracts served as templates in PCR amplification reactions using GoTaq polymerase (Promega) with amplification primers listed in Table 1. The PCR program followed the manufacturer’s recommendations with the primer-specific annealing temperatures of 57.5° for both loci. We subcloned the resulting amplicons into pJet1.2 plasmid (Thermo Fisher) and fragments obtained from purified colony PCR reactions were sequenced using M13 forward and reverse primers. The alignments of mutant and wild-type sequences were prepared manually. We considered an individual to bear a mutant genotype if at least two sequences represented mutant alleles (see Table 4).

### *In situ* Hybridization

Whole mount *in situ* hybridization (ISH) was carried out as described in detail by Minarik et al. (2017). We retrieved the putative sterlet homolog of *ripply3* from our pharyngula transcriptomes. The 317-bp long DNA template sequence was PCR amplified from sterlet cDNA (amplification primers in Table 1) and subcloned into pGEM T-Easy vector (Promega) by standard procedures. Acquisition of *foxd3* and *twist1* sterlet sequences was described by Stundl et al. (2020). ISHs using injected and wild-type embryos were performed in separate tubes though in parallel and under the precisely same conditions to avoid variations in probe penetration and signal development.

### Imaging

All photographs of sterlet embryos and larvae in PBS were taken with Olympus SZX12 stereoscopic microscope using z-stacking Deep Focus technology of QuickPhoto software (Promicra).

## Results

We used CRISPR/Cas9 system to induce insertions and deletions (indels) Figure 1D into protein-coding sequences of two sterlet genes; *tyr* and *shh*. *tyr* encodes the enzyme tyrosinase involved in melanin synthesis in vertebrates. Successful mutagenesis is expected to reduce pigmentation of sterlet larvae but should not affect other aspects of their normal development. On the other hand, *shh* is a developmental regulator of several organ systems, and its mutation is expected to have dramatic effects on early embryonic development of tissues and organ systems derived from neural crest cells and all three germ layers.

### *Tyr* Mutagenesis

We designed and synthesized two sgRNAs targeting two different exons of the sterlet homolog of human *Tyr1* - sgRNA three and four against exons 2 and 3, respectively, sequences of which allowed us to design sgRNAs according to our criteria (see Methods and Square et al., 2015; Square et al., 2020). First, we co-injected 50 one-cell sterlet embryos with a mix containing both guides at a total amount of ∼2.67 ng of sgRNA and 5.3 ng of Cas9 protein (in 1:2 wight ratio) per single embryo. The total amount was calculated to match the amount of guide RNA and Cas9 protein relative to the egg size successfully used in *Xenopus laevis* CRISPR/Cas9 mutagenesis (Square et al., 2015; Square et al., 2020). In total, 32 injected embryos were LRD positive (i.e., with the lysinated Rhodamine-dextran of the injection mix glowing under the fluorescent light) at the neurula stage, however, no larva showed reduction in pigmentation or other developmental malformation. We suspected that this could have resulted from concentration of each individual sgRNA being too low in this combined sgRNA mix and therefore we used the same total amount of the injection mix but with each sgRNA separately in the next experiment. Both these experiments produced albinos with the variable extent of pigment reduction and normal morphology lacking any visible morphological abnormalities. We visually assessed the extent of pigment reduction and scored the phenotypes to four classes at increments of 25% pigment reduction (Table 2). Injection with sgRNA three resulted in >70% partial or total albinism occurrence, while treatment with sgRNA four produced >50% partially or completely albinotic larvae. Mutagenesis with sgRNA three was more efficient in producing phenotypes with stronger pigment reduction, although the numbers of complete or near-complete albinos were similar between the guides. We recorded 25–33% mortality, which was similar to the uninjected dechorionated wild-type larvae raised to the same stage (Table 2) and to the mortality of embryos injected with non-functional sgRNA (14/50 = 28%).

**TABLE 2:** Summary of observed phenotypes of Δ*tyr* sterlet larvae.

	*tyr* sgRNA 3	*tyr* sgRNA 4
0–25% pigment reduction	29% (6/21)	50% (9/18)
25–50% pigment reduction	29% (6/21)	26% (5/18)
50–75% pigment reduction	33% (7/21)	11% (2/18)
75–100% pigment reduction	10% (2/21)	11% (2/18)
Injected in total	40	30
LRD positive embryos	21	18
Mortality	10 (25%)	10 (33%)

**FIGURE 2:**
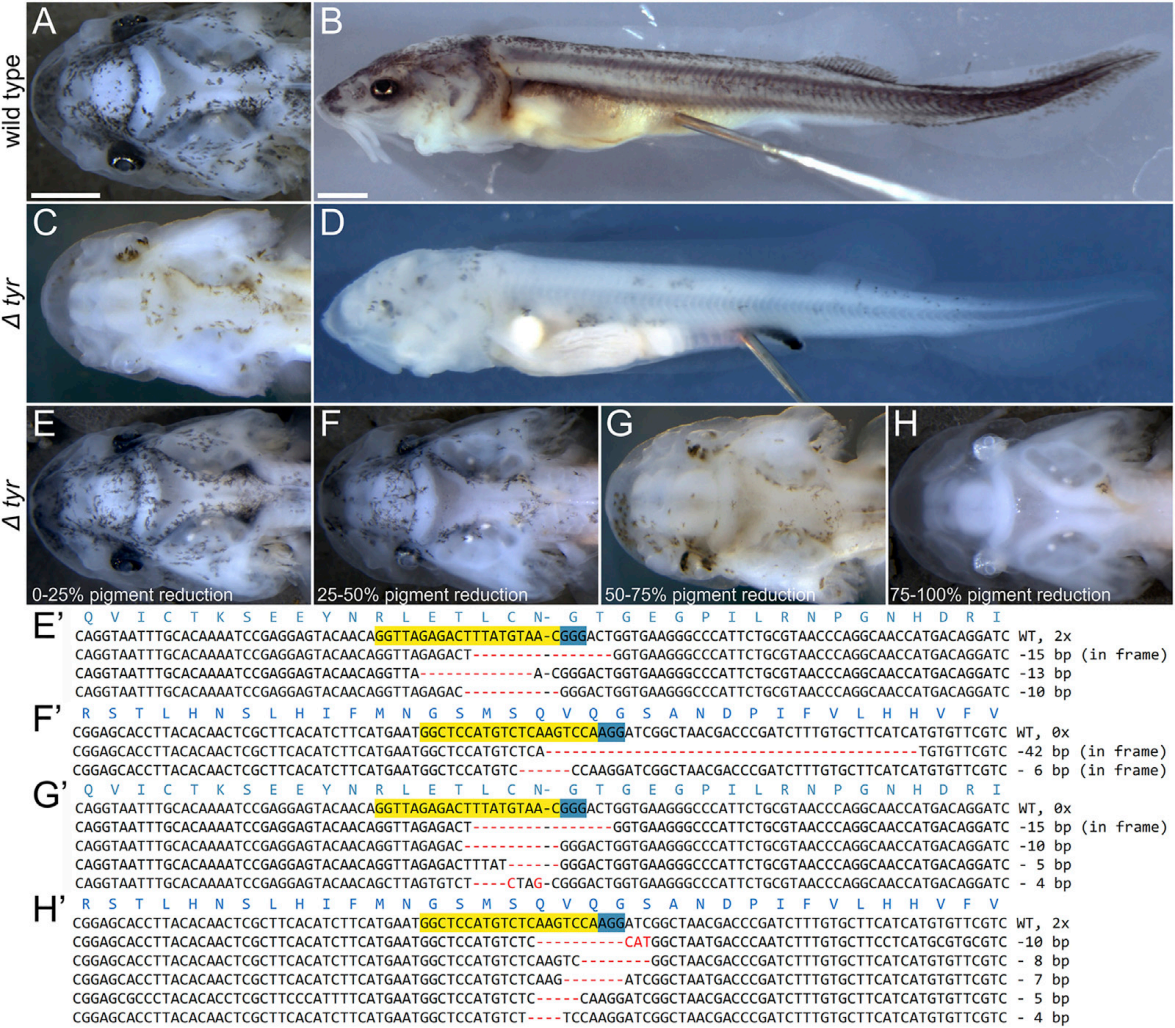
Phenotypes and genotypes of Δ*tyr* sterlet larvae. Dorsal **(A,C,E–H)** and lateral **(B–D)** views of ∼16 mm long larvae with anterior to the left. Wild type [WT; **(A,B)**] and representative mutant **(C,D)** larvae and four mutant classes **(E–H)** and their genotypes **(E′–H′)**. The CRISPR target sites and PAM on the forward sequences are highlighted in yellow and blue, respectively, and the red dashes and letters represent deletions, insertions, and polymorphisms relative to the WT sequence. Mutations in **(E′,G′)**, and **(F′,H′)** were introduced by *tyr* sgRNA three and sgRNA 4, respectively. Mutant and WT sequences were identified in all phenotype classes (see also Supplementary Figure S1), while sequencing of larvae portrayed on panels **(F,H)** only returned mutant sequences (as was the case of 3/11 mutant larvae). Scale bars (same in A + C + E-H and B + D) represent 1 mm.

We verified the disruption of the target *tyr* loci by genotyping selected individuals from each phenotype class (Figure 2, Supplementary Figure S1). We found mutant alleles in 7/10 and 4/7 genotyped larvae generated with sgRNA 3 and 4, respectively, and confirmed mutants in all phenotype classes. Approximately half of all obtained sequences were mutant. We observed 27 unique mutant alleles (out of 36 sequences), 22 of which had only deletions, one had only insertion and four had both deletions and insertions (Supplementary Figure S1). While we did not observe a correlation between the relative occurrence of WT vs. mutant alleles and severity of the observed phenotypes, it appears that the stronger phenotypes have more alleles with indels that cause frameshifts (Figure 2, Supplementary Figure S1).

### *Shh* Mutagenesis

**FIGURE 3:**
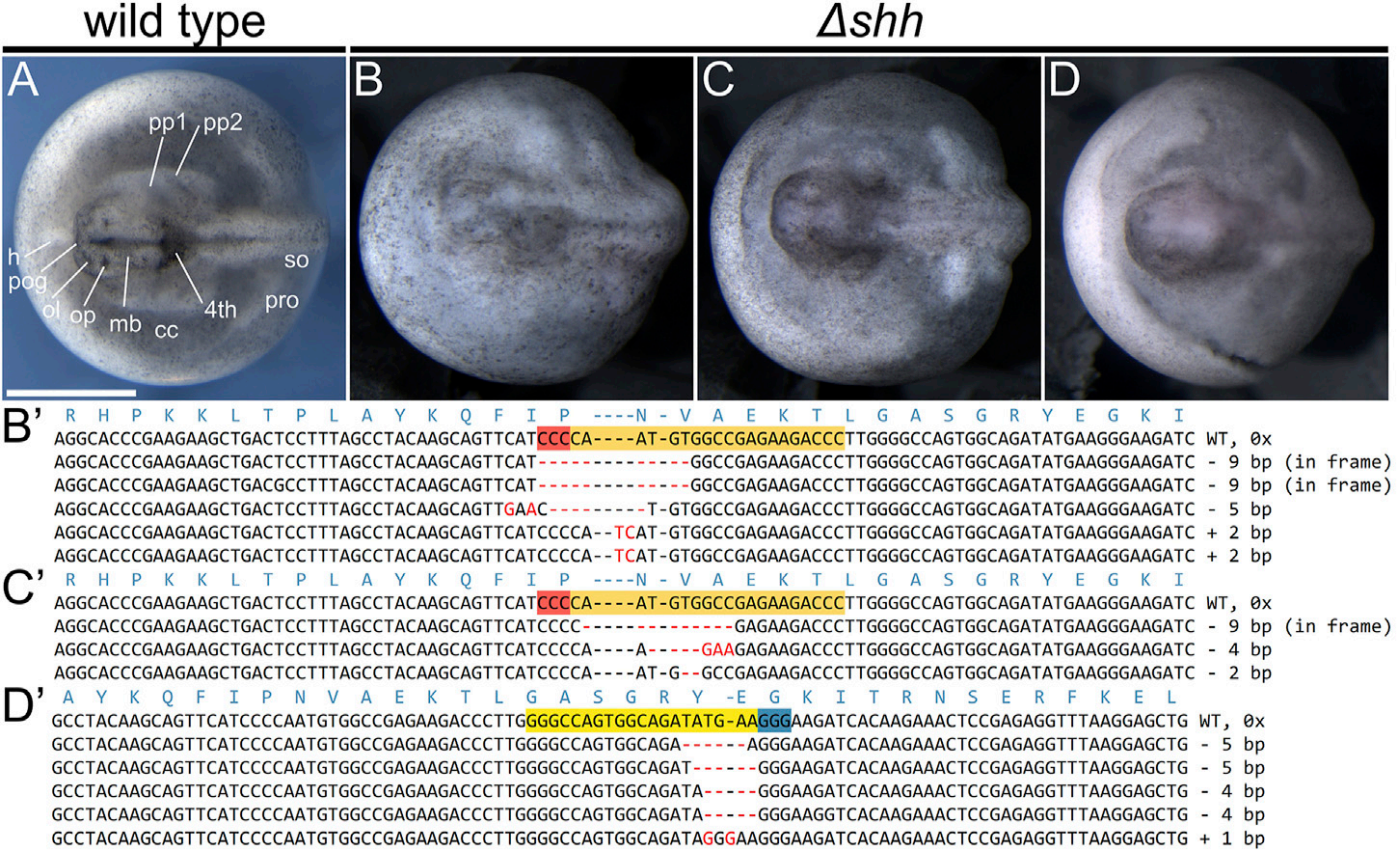
Phenotypes and genotypes of Δ*shh* sterlet larvae. Dorsal views of st. 28 larvae with anterior to the left. Wild type [WT; **(A)**] and mutant **(B–D)** embryos and their mutant alleles **(B′–D′)**. The CRISPR target sites are highlighted in orange with red PAM on the reverse strand sequences, while they are yellow with blue PAM on the forward strand. The red dashes and letters represent deletions, insertions, and polymorphisms relative to the WT sequence. Mutations in **(B′,C′)** were introduced using *shh* sgRNA 1, while the mutations in **(D′)** resulted from injections of *shh* sgRNA 2. No WT sequences were identified in any of the individuals showing *shh* phenotype (six genotyped individuals in total). The mutant embryo in **(B)** was scored as missing pre-oral gut, optic and olfactory placodes, forebrain, midbrain, part of the hindbrain, and pharyngeal pouches and with underdeveloped pronephros. The mutant embryo in **(C)** is missing pharyngeal pouches and has deformed forebrain and midbrain. The mutant embryo in **(D)** was identified as missing pharyngeal pouches and somites, the entire head is underdeveloped. Fourth—fourth brain chamber of hindbrain, cc—coelomic cavity, h—heart, mb—midbrain, ol—olfactory placode, op—optic placode, pog—pre-oral gut, pp—pharyngeal pouch, pro—pronephros, so—somites. Scale bar represents 1 mm.

Similar to our initial experiments with *tyr* mutagenesis, we first injected 50 single-cell sterlet embryos with a mix of both guides targeting *shh* locus with the total amount of ∼2.67 ng of sgRNA and 5.3 ng of Cas9 protein per embryo. We recorded relatively high mortality (36/50 = 72%), but the surviving LRD positive embryos (13; one was LRD negative) showed no discernable phenotypic effect. Next, we injected each sgRNA separately at the same total amount and sgRNA/Cas9 ratio. Due to high mortality in the initial experiment, we injected higher numbers with each guide in a separate mix - 191 with sgRNA 1 and 117 with sg RNA 2. We observed mortality of 34 and 38%, respectively (64/191 individuals in sgRNA one and 44/117 in sgRNA two injections) and obtained 93 and 57 LRD positive embryos that reached developmental st. 28. The embryos were severely affected with strong morphological deformations in almost all embryonic structures present at that stage (Table 3). The highest frequency phenotypes included missing pharyngeal pouches and pre-oral gut (up to 81%), followed by missing, reduced or malformed optic placode, not discernible heart, pronephros reduction and brain malformations. Such deformations were consistent with patterns of *shh* gene expression observed during the sterlet embryonic stages preceding the analyzed stages (Figures 3A–C). Only 7–10% of injected LRD positive individuals appeared to have no visible phenotype (9/93 and 4/57 for sgRNA 1 and 2, respectively). We did not observe any of the described phenotypes in the control embryos and their mortality was similar to the mortality in *tyr* mutagenesis experiment.

**TABLE 3:** Summary of observed phenotypic effects of Δ*shh* sterlet embryos.

A)	*shh* sgRNA 1		*shh* sgRNA 2	
	Missing/not visible	Reduced/malformed	Missing	Reduced/malformed
Heart	23% (21/93)	—	16% (9/57)	—
Pre-oral gut	47% (44/93)	—	46% (26/57)	—
Olfactory placode	13% (12/93)	8% (7/93)	12% (7/57)	9% (5/57)
Optic placode	22% (20/93)	34% (32/93)	23% (13/57)	30% (23/57)
Phar. pouch 1	73% (68/93)	—	81% (46/57)	—
Phar. pouch 2	69% (64/93)	—	68% (39/57)	—
Forebrain	9% (8/93)	24% (22/93)	7% (4/57)	21% (12/57)
Midbrain	6% (6/93)	17% (16/93)	7% (4/57)	12% (7/57)
Hindbrain	6% (6/93)	18% (17/93)	4% (2/57)	14% (8/57)
Somites	9% (8/93)	—	5% (3/57)	—
Pronefros	—	33% (31/93)	—	26% (15/57)
100% Healthy embryos	10% (9/93)		7% (4/57)	
**B)**	**missing**	***Shh* mutant**	**reduced**	
*foxd3* ISH				
-Trigeminal NC	0/3		3/3	
-Hyoid NC	0/3		3/3	
-Branchial NC	0/3		3/3	
100% Healthy embryos		0/3		
*twist1* ISH				
-Maxillary NC	1/14		3/14	
-Mandibular NC	4/14		3/14	
-Hyoid NC	1/14		9/14	
-Branchial 1 NC	1/14		7/14	
-Branchial 2 NC	5/14		5/14	
100% Healthy embryos		3/14		
*ripply3* ISH				
-Pharyngeal pouch 1	4/7		3/7	
-Pharyngeal pouch 2	0/7		7/7	
-Pharyngeal pouch 3	4/7		3/7	
100% Healthy embryos		0/7		

**FIGURE 4:**
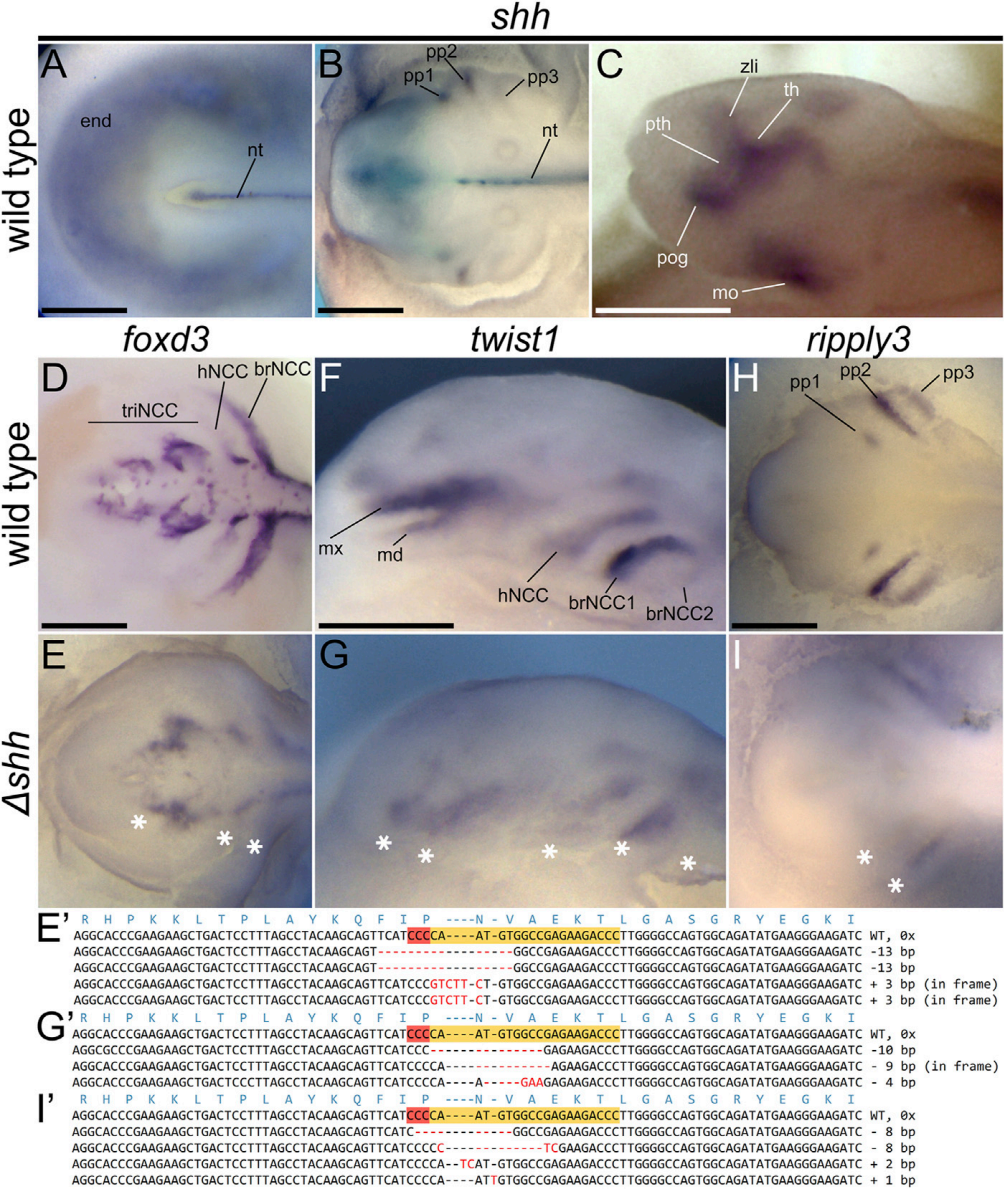
*shh* expression patterns and Δ*shh* sterlet embryos at st. 28 showing defects in expression patterns of *foxd3*, *twist1*, and *ripply3*. Dorsal **(A,B,D,E,H,I)** and lateral **(C,F,G)** views with anterior to the left. Patterns of *shh* expression in various morphological structures of the wild-type sterlet embryos at stages 23 **(A)**, 28 **(B)**, and 30 **(C)**. *foxd3* marks neural crest cells (NCCs) at early stages of migration, *twist1* marks NCC at later migration stages, and *ripply3* is expressed in developing pharyngeal pouches. Compare with Supplementary Figure S3 to see the observed variation in expression patterns in both wild-type and mutant individuals. The sequences show the reverse DNA strands with *shh* sgRNA one CRISPR target sites highlighted in orange with red PAM site. The red dashes and letters show indels and polymorphisms relative to the WT sequence. Asterisks indicate missing or reduced expression in mutant embryos. brNCC—branchial stream of NCCs, hNCC—hyoid stream of NCCs, md—mandibular stream of trigeminal stream of NCCs, mo—mouth, mx—maxillary stream of trigeminal stream of NCCs, nt—notochord, pog—pre-oral gut, pth—prethalamus, pp—pharyngeal pouch, th—thalamus, triNCC—trigeminal stream of NCCs, zli—zona limitans intrathalamica. Scale bars represent 0.5 mm.

Next, we analyzed effects of *shh* disruption on expression patterns in injected sterlet embryos by ISH. We examined three genes known to be involved in development of some of the affected structures at early embryonic stages: *foxd3* marking NCCs at early migration stage, *twist1* NCCs at later stages of migration, and *ripply3* in forming pharyngeal pouches (Figure 4, Supplementary Figure S3). We found that all these markers were reduced in the injected sterlets at st. 28. *foxd3* showed a reduction in the size of all three streams of migrating NCC (trigeminal, hyoid, and branchial), *twist1* expression was reduced in all later NCC populations (maxillary, mandibular and branchial) and even entirely missing in some embryos. Only three out of 14 hybridized embryos appeared to have a wild-type expression pattern of the NCC markers. *ripply3* ISH confirmed our observation of the strong effect of *shh* mutation on pharyngeal pouch formation in putative *shh* mutants; all seven analyzed embryos had either missing or reduced expression domains (Table 3).

To verify mutagenesis in *shh* locus in analyzed sterlet embryos, we genotyped selected individuals injected by both sgRNAs, including those used for ISHs. All 33 obtained sequences had indels and consequently all nine genotyped individuals were confirmed mutants. We identified 21 unique alleles in total, five of which only showed insertions, 12 of them contained deletions and four had both insertions and deletions (Table 4). The majority of the observed mutations caused frameshifts; these were observed in at least half of all obtained sequences of each mutant individual. Only 8/26 sequences of mutations introduced by sgRNA one were in-frame (Figure 4, Figure 3, Supplementary Figure S2, Table 4).

**TABLE 4:** Summary of sterlet Δ*tyr* larvae and Δ*shh* embryos genotyping. A) all obtained sequences from all genotyped individuals pooled together, B) unique alleles from all genotyped individuals pooled together, C) all genotyped individuals. The denominator numbers in A represent all obtained sequences from all individuals genotyped for a respective locus, and similarly in B, the denominators show the total number of mutant alleles obtained from sequencing all individuals. In C, the denominators indicate the total numbers of genotyped individuals for each respective locus. Mutation and frameshift rates are calculated for each row, i.e. they represent percentages of mutant sequences and unique alleles from all sequences obtained by genotyping all individuals pooled together in A and B, respectively, while they show percentages of genotyped individuals with mutant/frameshifted genotypes in C.

	Mutant	WT	Frameshift	Mutation rate	Frameshift rate (%)
A) Sequences					
*tyr* sgRNA 3	21/46	25/46	11/21	46%	52
*tyr* sgRNA 4	15/30	15/30	8/15	50%	53
*shh* sgRNA 1	26/26	0/26	18/26	100%	69
*shh* sgRNA 2	7/7	0/7	7/7	100%	100
B) Unique alleles					
*tyr* sgRNA 3	14/21	—	7/14	—	50
*tyr* sgRNA 4	13/15	—	8/13	—	62
*shh* sgRNA 1	15/26		11/15		73
*shh* sgRNA 2	6/7	—	6/6	—	100
C) Individuals					
*tyr* sgRNA 3	7/10	3/10	6/7	70%	86
*tyr* sgRNA 4	4/7	3/7	2/4	57%	50
*shh* sgRNA 1	7/7	0/7	7/7	100%	100
*shh* sgRNA 2	2/2	0/2	2/2	100%	100

## Discussion

### Sterlet is Highly Amenable to CRISPR/Cas9 F0 Mutagenesis

Here we report the application of a CRISPR/Cas9 mutagenesis strategy previously optimized in the sea lamprey (Square et al., 2015) and African clawed frog (Square et al., 2020), to the sterlet. Previous reports using TALENs and CRISPR/Cas9 suggested sterlet zygotes will tolerate injection with proteins and nucleic acids and were amenable to gene targeting (Chen et al., 2018; Baloch et al., 2019). We found that our optimized protocol introduced biallelic mutations in the targeted loci at relatively high efficiency and yielded consistent and reproducible phenotypes. As expected, targeting of the gene *tyr*, resulted in larval albinism with no other discernible developmental defects. These mutants have similar mortality as their uninjected wild type siblings. The visual scoring of the larval phenotypes is straightforward, suggesting that *tyr* targeting could serve as a robust positive control in future studies. In contrast to *tyr* disruption, mutating the pleiotropic developmental regulator *shh* caused defects in a wide range of embryonic and larval tissues and structures. Interestingly, both sgRNAs used to target *shh* resulted in all injected individuals displaying defects consistent with the known roles of *shh* in vertebrate development. This demonstrates that determining the function of developmental regulators, even highly pleiotropic ones, is readily achievable in sterlet. Arguably, in CRISPR/Cas9 sterlet F0 mutants it could be more challenging to detect and score very subtle and less conspicuous phenotypes than in models with stable inbred lines.

Though sterlet is not well suited as a laboratory-propagated model organism, several features of its natural history make it a strong candidate for routine genetic analyses of “F0” mosaic mutants. Along with other sturgeon species, it is a frequently farmed fish, whose eggs can be seasonally obtained in large quantities. The eggs are relatively large and can be easily manipulated, dechorionated, and injected with just forceps and also simple hand-held and mouth operated injector. The embryos and larvae thrive in simple aquaria with no special care requirements beyond clean oxygenated water and a constant temperature of 15–17°C. Several common laboratory methods have also already been optimized in the species including histological sectioning, microCT scanning, RNA *in situ* hybridization, lineage tracing following injections, and immunohistochemistry (Minarik et al., 2017; Stundl et al., 2020).

Other features of sterlet, and acipenseriform fishes in general, were likely important for the successful application of our CRISPR/Cas9 mutagenesis strategy. Sterlet eggs are rich in yolk that is distributed throughout the egg (Dettlaff et al., 1993). The eggs of lamprey and frog, which are highly amenable to CRISPR/Cas9 mutagenesis (Square et al., 2015; Square et al., 2020), are similarly rich in yolk. Since as in frog, the sterlet egg yolk is slightly more concentrated on the vegetal pole, the animal pole with more yolk-free cytoplasm and nucleus are better accessible for the injected CRISPR/Cas9 mix. Therefore, we tried to target the animal pole, usually conveniently situated on the top of the embryo. However, we suspect that because acipenseriforms undergo holoblastic cleavage (Dettlaff et al., 1993; Ostaszewska and Dabrowski, 2009) injecting any part of the zygote’s cytoplasm would likely work well. Similar to frog, but unlike lamprey, sturgeon eggs have relatively thick envelope. While this does not prevent injection of the embryo with a capillary needle, we preferred to remove the thick outer layer with forceps to better see the injected droplet size and target the animal pole (frog eggs jelly membranes are removed by cysteine treatment; Sive et al., 2000). Due to the similarities between the zygotes of sterlet and those of lamprey with frog, we decided to use approximately the same concentration of injected RNA and protein, and approximately the same relative volume of injection solution. In practical terms, this means we injected a droplet of CRISPR/Cas9 injection mixture with a diameter approximately 1/7 that of the zygote diameter. Because this strategy works well in sterlet, lamprey, and African clawed frog, we suspect a 1/7 size droplet would yield good results in any vertebrate with isolecithal, mesolecithal, or polylecithal eggs displaying holoblastic cleavage. In contrast, zygotes of embryos with meroblastic cleavage (e.g., Takeuchi et al., 2009), such as zebrafish and other teleost fish, appear to tolerate lower amounts of RNA/protein (Hwang et al., 2013; Jao et al., 2013), and lower volumes of injection mix. For such embryos, the injected volume should likely be adjusted based on the size of the blastodisc rather than the entire embryo.

Recent publication of high-quality sterlet genome (Du et al., 2020) should allow the effective design of highly specific sgRNAs. We initially only searched our embryonic transcriptomes for potential off-target sequences. However, additional searches of the new sterlet genome, including non-coding sequences, did not identify any other off-target sequences. When designing sgRNAs, we found that approximately one third of the initially selected guide sequences conformed with off-target sequences according to the criteria described in Methods. When performing CRISPR/Cas9 mutagenesis in sea lamprey and African clawed frog we found that using at least two different sgRNAs per gene, and injecting them separately, is the best strategy for producing verifiable and consistent phenotypes and control for any defects caused by off-target lesions. We observed highly consistent phenotypes and no indication of off-target effects in sea lamprey after mutating more than 20 different genes (Square et al., 2020). The results presented here suggest a similar level of specificity and consistently can be expected in sterlet.

We only observed a moderate disparity in the efficiency of individual sgRNAs. While the mutation rates varied between *tyr* and *shh* loci, they were very similar between the guides targeting the same gene (Table 4), with subtle differences recorded among mutant categories. This contrasts with sea lamprey and other species, in which some degree of variation was observed among different guides targeting the same genes (Hsu et al., 2013; Square et al., 2015). We expect that injecting more eggs and guides would result in higher variation in sgRNA efficiency in sterlet as well. On the other hand, as we observed in sea lamprey, mutations causing frameshifts in coding DNA sequences correlated with more severe phenotypes in sterlet as well.

To confirm successful mutagenesis, we also genotyped representative individuals from each phenotypic class. While PCR fragment length is often used to tell whether an individual harbors mutant alleles, we find that this method often produces ambiguous results. We thus chose to clone and sequence genomic fragments including the target sequence and approximately ∼200 bp of flanking sequence on each side. Sequencing of phenotypic mutants and controls, confirmed highly efficient mutagenesis with all tested sgRNAs, with the frequency and type of alleles correlating with phenotype. While even two to three of sequences per individual are sufficient to confirm a baseline level of mutagenesis, we find sequencing ten or more clones gives a view of allelic composition that usually correlates with phenotypes, and also suggests the efficiency of the sgRNA. We also found that, as with lamprey embryos (York et al., 2017), fixed *in situ* hybridized sterlet embryos can be successfully genotyped if they are not post-fixed with formaldehyde for extended periods of time (see also Square et al., 2020).

### Prospects for Adapting CRISPR/Cas9 F0 Mutagenesis to Other “Basal” Fishes

The efficiency, precision, and ease of use of CRISPR/Cas9 mutagenesis in sterlet, lamprey, and African clawed frog strongly suggest this method can be successfully applied to other non-teleost fish. Besides sterlet, several other sturgeon species are currently farmed. While sterlet is probably the most suitable model for developmental studies due to its smaller size and monoploid genome, several other species with different levels of polyploidy (Havelka et al., 2016) could be attractive for studies of genome evolution. While polyploidy necessarily complicates targeted gene mutagenesis by requiring simultaneous disruption of homeologs to yield a valid loss-of-function, the efficiency of CRISPR/Cas9 can be exploited to overcome this issue. We and others have found that targeting conserved homeolog sequences, and/or using multiple sgRNAs can yield strong phenotypes in the allotetraploid African clawed frog (Wang et al., 2015; Square et al., 2020). Gene manipulation in larger species of sturgeons can potentially be useful in sturgeon food production industry when targeting genes involved in growth, immunity, or egg production.

American paddlefish (*Polyodon spathula*) is morphologically unusual acipenseriform relative of sturgeons. Like sturgeon, it is also frequently farmed, and its embryos have been used in several evolutionary developmental studies (e.g. Davis et al., 2007; Modrell et al., 2011). There are currently no published reports of CRISPR/Cas9 mutagenesis in paddlefish. However, high similarity of paddlefish and sterlet embryos, and successful previous experiments involving dye injections at later embryonic stages suggest that CRISPR/Cas9 mutagenesis could be highly effective in paddlefish. Unfortunately, collecting of early paddlefish embryos appears to be more challenging than sterlet in part because of the shorter paddlefish spawning season (Modrell et al., 2017).

Besides acipenseriforms, three non-teleost fish lineages have extant members that have been utilized in comparative embryological studies and have published genomes - bichirs (Polypteriformes), gars (Lepisosteiformes), and bowfins (Amiiformes) (Askary et al., 2016; Braasch et al., 2016; Minarik et al., 2017; Stundl et al., 2019; Funk et al., 2020; Stundl et al., 2020; Thompson et al., 2021; Bi et al., 2021). While bichirs and bowfins are evolutionarily very attractive and informative species with unique morphologies, the main challenge in targeted mutagenesis in these organisms is obtaining zygotes for injection. Bichirs reproduce in aquaculture settings and can occasionally spawn in home aquaria. However, their mating is secretive and usually occurs at night. Thus, collection of zygotes requires constant observation, which could disturb the animals and prevent spawning. Once collected, though, bichir embryos are relatively sturdy, and can be manipulated and injected with vital dyes much like African clawed frog (Stundl et al., 2019). Bowfin eggs are typically only collected in the wild, and their zygotes are thus less accessible than those of bichir (Funk et al., 2020). Fortunately, decapsulating of later bowfin embryonic stages is relatively easy, so it is likely that early embryos could be injected if obtained (Brent Hawkins, personal communication). Cleavage of bowfin embryos is intermediate between the holoblastic mode of most non-teleost fishes and the meroblastic cleavage of teleosts, with reduced cleavage on the vegetal hemisphere (Cooper and Virta, 2007). This would suggest that if bowfin zygotes were collected, a smaller injection volume might be needed for embryos to survive. Besides sturgeons, perhaps the most promising non-teleost fish candidates for CRISPR/Cas9 mutagenesis are gars. Several species are kept and propagated at fish farms, and like in sturgeons, large quantities of eggs can be seasonally obtained in a controlled fashion *in vitro* fertilization (Braasch et al., 2014 in spotted gar; Minarik et al., 2017 in tropical gar). Subsequent manipulation with zygotes and raising the offspring is not complicated and the similarity of gar and sturgeon eggs should make injecting relatively straightforward. One potentially confounding factor worth mentioning is that gar embryos, like those of lamprey, lack maternal pigmentation, making it difficult to discern when cleavage begins (Comabella et al., 2014).

While other non-teleost ray-finned fish species are likely well suited to CRISPR/Cas9 F0 mutagenesis, two key groups of early-diverging vertebrates, the chondrichthyans, and hagfish, are unlikely to be amenable to this method, at least as described here. Representatives of both groups have been used in developmental studies in the last 2 decades and have provided some fundamental insights into the developmental bases of vertebrate evolution (Ota et al., 2008; Gillis et al., 2009; Oisi et al., 2013; Gillis and Tidswell, 2017). Embryos of both chondrichthyan clades; elasmobranchs (sharks, rays, and skates) and holocephalans (chimaeras) can be collected seasonally in the wild or in public aquaria, sometimes in the dozens (Gillis, 2011). However, zygotes that could potentially be injected are extremely difficult to obtain and, so far, have not been collected in the numbers needed for reproducible gene disruption. The logistics of hagfish embryology are even more challenging. The few classical reports of hagfish embryology have been based on the scarce embryonic material collected in late 19th century. Only recently have substantial numbers of live hagfish embryos been collected and analyzed (Ota and Kuratani, 2006; Kuratani and Ota, 2008; Ota and Kuratani, 2008). To our knowledge, none of these embryos have ever been successfully injected as zygotes.

## Conclusion

Based on the results reported here, and previous experiences of ourselves and others, we posit that our CRISPR/Cas9 mutagenesis strategy will work well in any vertebrate with zygotes that are easily accessed and can tolerate microinjection. In such animals, we expect the main variable determining success will be injection droplet size, which will likely need to be adjusted according to the egg size and yolk distribution. Current limitations seen in chondrichthyans may be circumvented with techniques that allow mass transfection of cells at later embryonic stages, such as electroporation or viral vectors.

## Data Availability

The original contributions presented in the study are included in the article/Supplementary Material, further inquiries can be directed to the corresponding author.
