# Supplementary material for: Efficient CRISPR Mutagenesis in Sturgeon Demonstrates Its Utility in Large, Slow-Maturing Vertebrates
Source: Front Cell Dev Biol. 2022 Feb 10;10:750833. doi: 10.3389/fcell.2022.750833 (PMC8867083; doi:10.3389/fcell.2022.750833)
Supplement: Supplementary file 1 [file DataSheet1.PDF]

Efficient CRISPR mutagenesis in sturgeon demonstrates its utility in large, slow-maturing vertebrates

Jan Stundl, Vladimír Soukup, Roman Franěk, Anna Pospisilova, Viktorie Psutkova, Martin Pšenička, Robert Cerny, Marianne E. Bronner, Daniel Meulemans Medeiros, David Jandzik

Supplementary Material

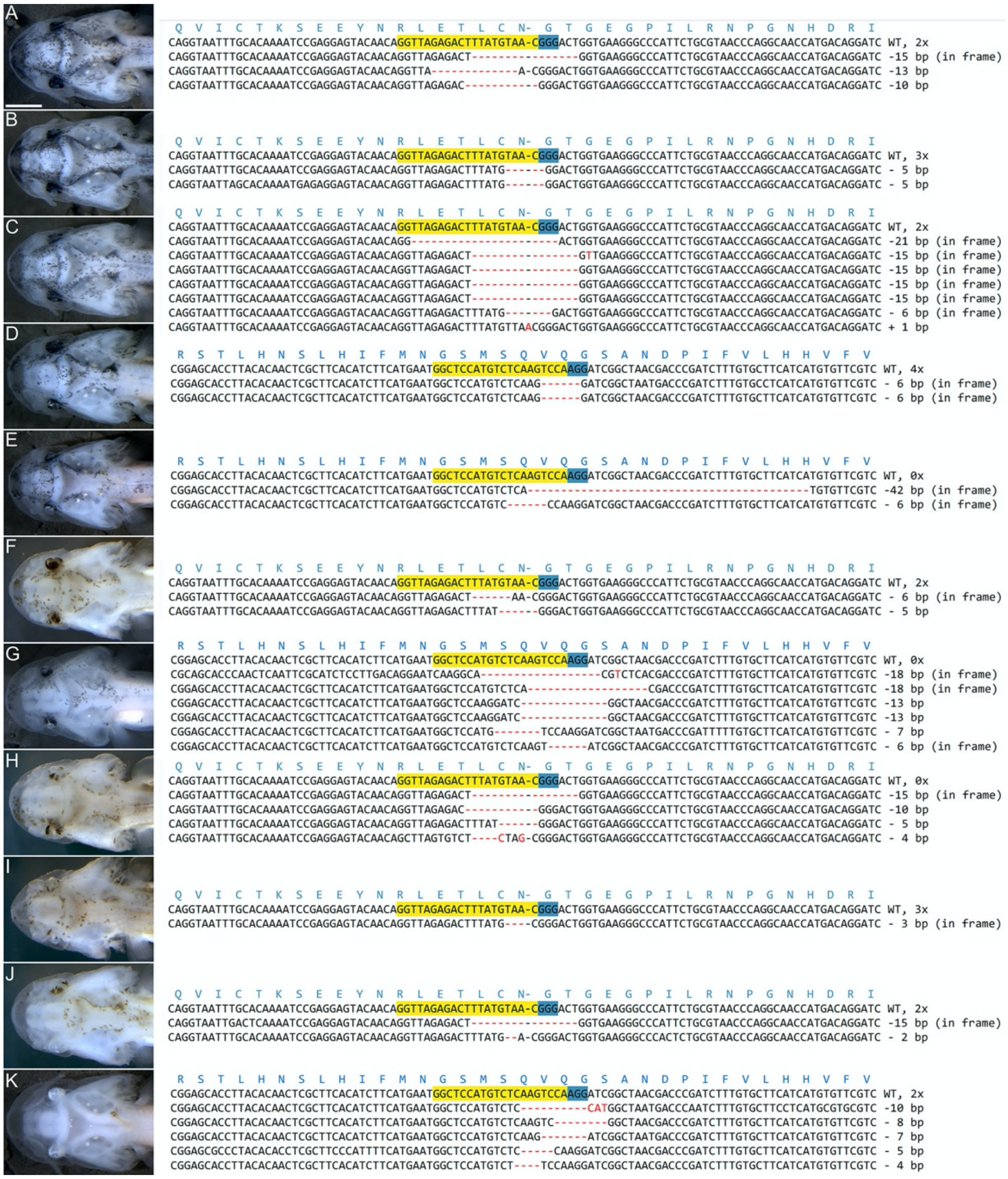

Supplementary Figure 1. Phenotypes and genotypes of all genotyped Δ*tyr* sterlet larvae. Dorsal views of ~16 mm long larvae with anterior to the left. The CRISPR target sites are highlighted in yellow with blue PAM on forward strand of *tyr* sequences and the red dashes and letters represent indels and polymorphisms relative to the WT sequence. Mutations in individuals on panels A-C, F, H, I were introduced by *tyr* sgRNA 3 and mutations in D, E, G, J by *tyr* sgRNA 4. Mutant and WT sequences were identified in all phenotype classes, while sequencing of larvae portrayed on panels E, G, H only returned mutant sequences (3/11 total mutant larvae). Individuals were scored as follows: A-C with 0-25%; D, E with 25-50%, F-J 50-75%, and I 75-100% pigment reduction. Scale bars represent 1 mm.

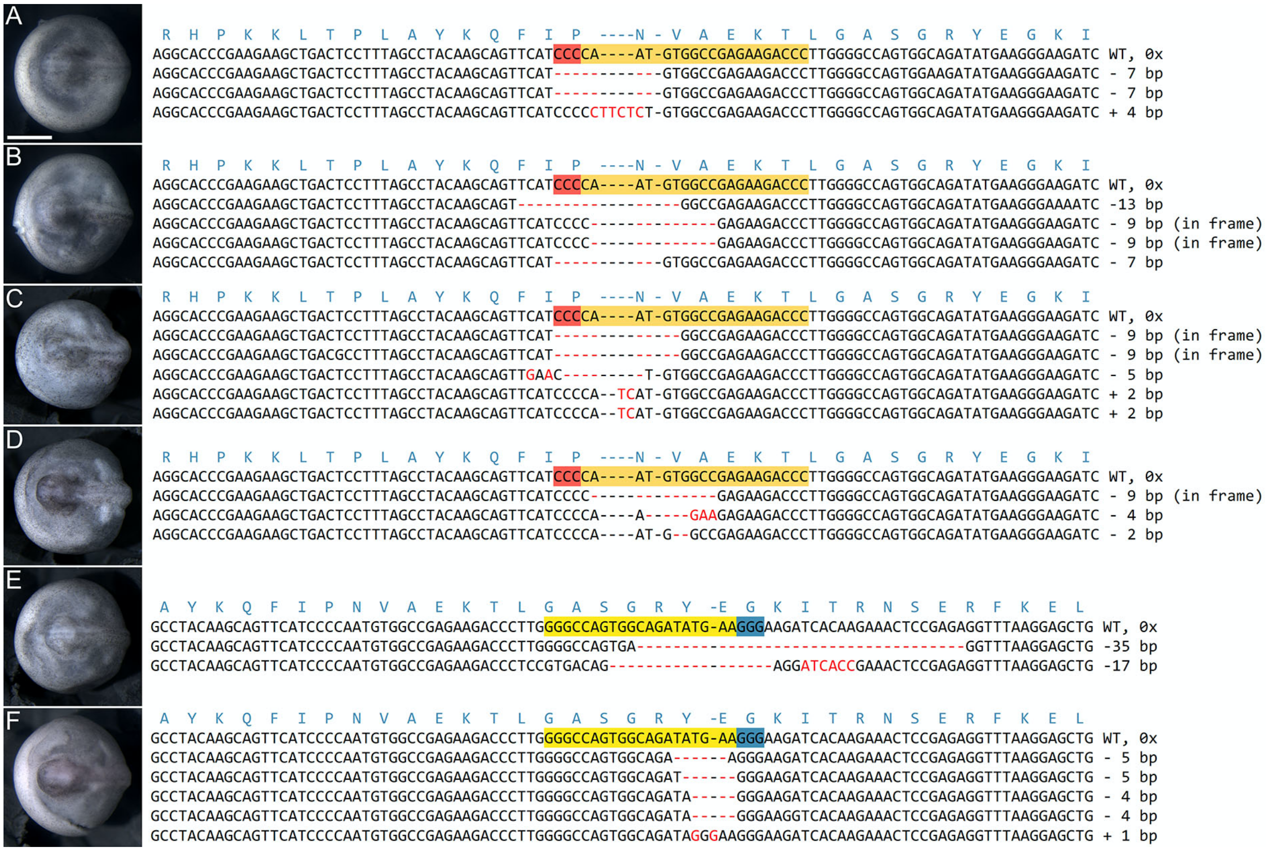

Supplementary Figure 2. Phenotypes and genotypes of all genotyped Δ*shh* sterlet larvae. Dorsal views of st. 28 larvae with anterior to the left. The CRISPR target sites are highlighted in orange with red PAM on reverse strand sequence, while they are yellow with blue PAM on forward strand sequences. The red dashes and letters represent indels and polymorphisms relative to the WT sequence. Mutations in A-D were introduced using *shh* sgRNA 1, while the mutations in E-F resulted from injections of *shh* sgRNA 2. No WT sequences were identified in any of the individuals showing *shh* phenotype. Scale bar represents 1 mm.

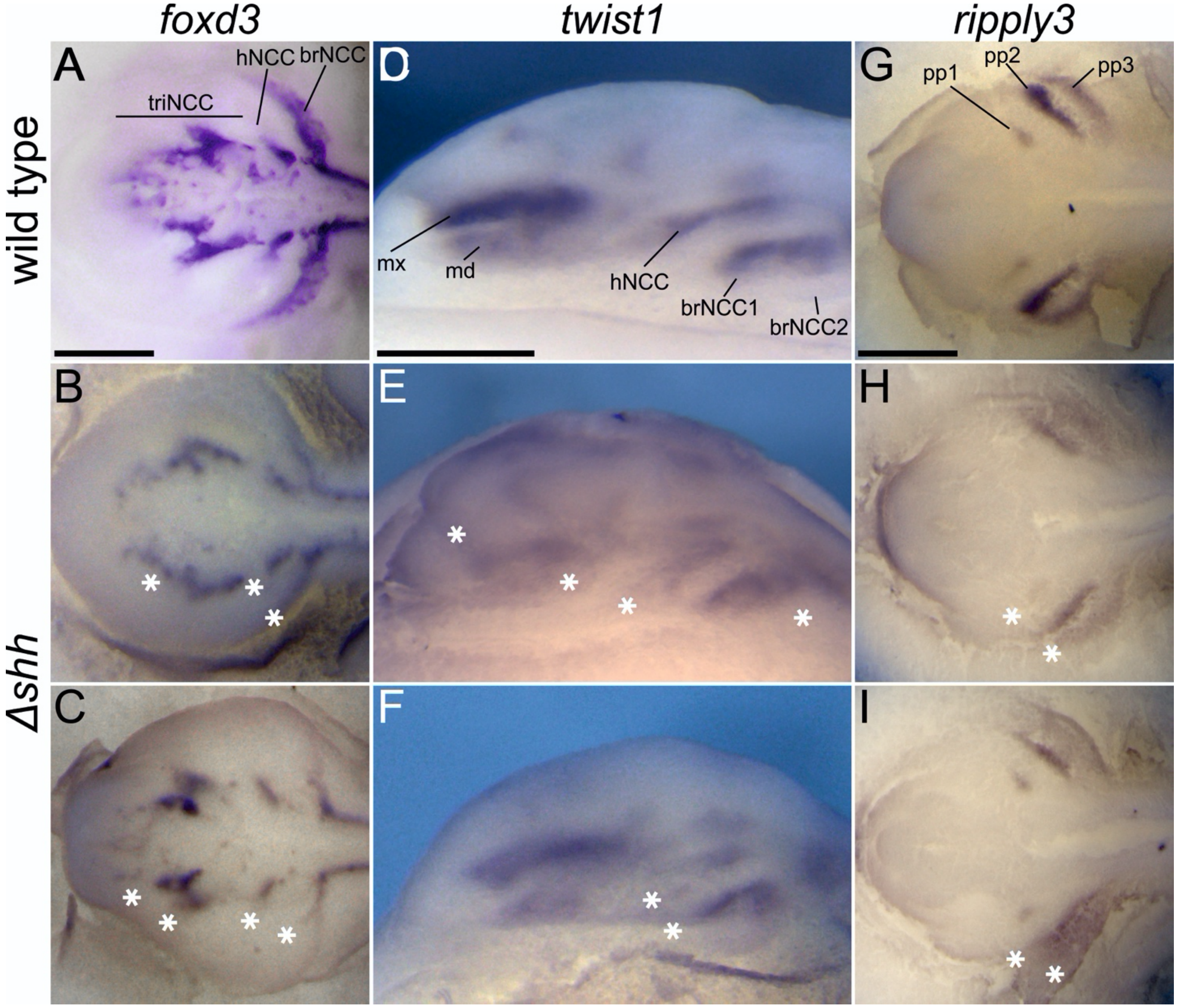

Supplementary Figure 3. Δ*shh* sterlet embryos at st. 28 showing defects in expression patterns of *foxd3*, *twist1*, and *ripply3*. Compare with Fig. 3 to see the observed variation in expression patterns in both wild-type and mutant individuals. Dorsal (A-C, G-I) and lateral (D-F) views with anterior to the left. *foxd3* marks neural crest cells (NCCs) at early stages of migration, *twist1* marks NCC at later migration stages, and *ripply3* is expressed in developing pharyngeal pouches. Asterisks indicate missing or reduced expression in mutant embryos. brNCC - branchial stream of NCCs, hNCC - hyoid stream of NCCs, md - mandibular stream of trigeminal stream of NCCs mx - maxillary stream of trigeminal stream of NCCs, pp - pharyngeal pouch, triNCC - trigeminal stream of NCCs. Scale bars represent 0.5 mm.
